# A Rare Variation of High Division of the Sciatic Nerve and Associated Neuromuscular Variations in the Gluteal Region

**DOI:** 10.7759/cureus.37187

**Published:** 2023-04-05

**Authors:** Ayush Amlan, Abdul W Ansari, Alka V Bhingardeo, Mrudula Chandrupatla, Shambhavi Bojja

**Affiliations:** 1 Anatomy, All India Institute of Medical Sciences, Bibinagar, Hyderabad, IND

**Keywords:** superior gemellus, posterior cutaneous femoral nerve, common peroneal nerve, tibial nerve, sciatic nerve

## Abstract

Variations in the anatomical division of the sciatic nerve are not uncommon. In this case report, we are presenting a rare variation of the sciatic nerve in relation to the superior gemellus and the presence of anomalous muscle. To the best of our knowledge, the anomalous communicating branches of the posterior cutaneous femoral nerve with tibial and common peroneal nerve and the presence of an anomalous muscle originating from the greater sciatic notch and inserting at ischial tuberosity have not been reported yet in the literature. This anomalous muscle found can be named as 'Sciaticotuberosus' after its origin and insertion.

Such variations hold clinical significance as they may contribute to piriformis syndrome, coccydynia, non-discogenic sciatica, and popliteal fossa block failure leading to local anesthesia toxicity and blood vessel traumatization. The current classifications of division of the sciatic nerve are based on its relation to the piriformis muscle. In our case report, the variation of the sciatic nerve in relation to the superior gemellus suggests the need for the revision of current classification systems. Category-like division of the sciatic nerve in relation to the superior gemellus muscle can be added.

## Introduction

Sciatic nerve is the thickest and longest nerve in the body [1-3]. Sciatic nerve is also known as the ischiadic nerve [3]. It is the branch of the sacral plexus formed by ventral rami L4 to S3 roots. It leaves the pelvis by passing through the greater sciatic foramen, usually below the piriformis muscle, and enters the gluteal region [3]. It is accompanied medially by the posterior cutaneous femoral nerve and the inferior gluteal artery [3]. Muscular branches of the nerve are distributed to all the hamstrings. Sensory branches supply the whole tibial and foot areas with the exception of the anteromedial tibial region and medial margin of the foot supplied by the saphenous nerve [3]. Sciatic nerve further divides into two terminal branches called tibial and common peroneal nerves. The Tibial component is formed by a ventral branch of the ventral rami of L4 to S3 spinal nerves, while common peroneal component is formed by the dorsal branch of the ventral rami of L4 to S2 spinal nerves [3]. The point of division of the sciatic nerve into the tibial and the common peroneal nerve is variable. The common site is at the junction of the middle and lower thirds of the thigh, near the apex of the popliteal fossa. The division may occur at any level from the pelvis to the superior angle of the popliteal fossa [1-6].

Variations in the anatomical division of the sciatic nerve from the pelvis to the popliteal fossa may contribute to piriformis syndrome, sciatica, coccydynia, and muscle atrophy [3,6]. The manifestations vary depending upon the level of division of the nerve into tibial and common peroneal [7-10]. The sciatic nerve holds great clinical significance as complete palsy of the sciatic nerve results in a flail foot and severe difficulty in walking [11-12]. The nerve is vulnerable to posterior dislocation of the hip, external compression, and misplaced therapeutic injections into the gluteus maximus [13-17].

In this case report, we are presenting a rare unilateral variation of the sciatic nerve in relation to the superior gemellus and the presence of anomalous muscle. This is not mentioned anywhere in our literature search, to the best of our knowledge. The knowledge of these variations is of utmost importance to surgeons for the planning of surgeries around the sciatic nerve.

## Case presentation

In a routine dissection of an approximately 50-year-old female cadaver, we found a unique unilateral variation of the right sciatic nerve. This is a case report about variation found in routine cadaveric dissection so exempted from ethical permission.

**Figure 1:**
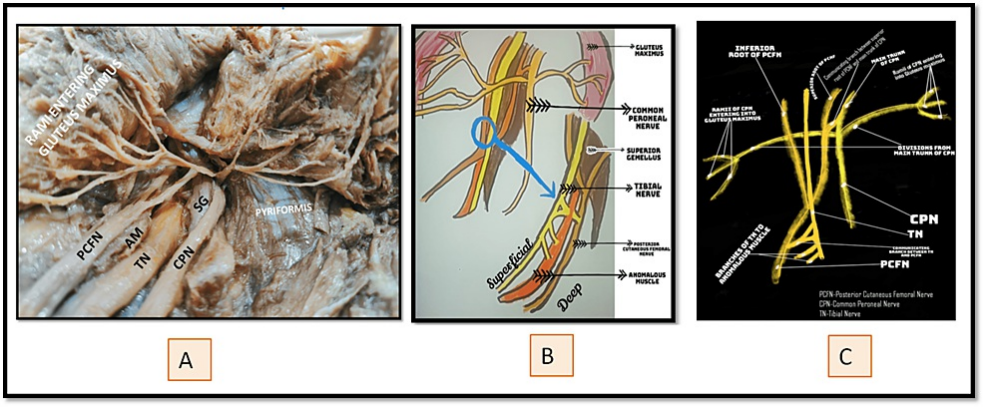
Images to show the nerve variation (A) Dissection image of variation, (B) Pictorial presentation of the same dissection image depicted in image A, (C) Schematic representation of nerves and their communications depicted in dissection image A. (B) and (C) are drawn by the Authors and are pictorial and schematic presentations of the same dissection image for better understanding of readers. They are not copied from any source, book, or publication. PCFN: Posterior Cutaneous Femoral Nerve; AM: Anomalous Muscle; TN: Tibial Nerve; CPN: Common Peroneal Nerve; SG: Superior Gamellus

After reflection of skin and abundant gluteal fat pad and reflecting Gluteus maximus muscle towards its origin, to our surprise, it was noticed that there was no sciatic nerve bifurcating into the tibial and common peroneal nerve at the apex of the popliteal fossa. Instead, the tibial and common peroneal nerve seemed to arise from lumbosacral plexus, which was confirmed on deep dissection. The nerves were present in relation to Superior gemellus muscle, occupying either side of the muscle. On deep dissection, we found that a common nerve trunk gave rise to three divisions superior, middle and inferior. The middle division continued as common peroneal nerve. Rest two divisions divided into so many rami and supplied gluteus maximus muscle. Inferior division gave a communicating branch to Posterior Cutaneous Femoral Nerve (PCFN). We found PCFN of thigh arising from two roots- "superior root and inferior root” as shown in Figure 1A. The posterior cutaneous femoral nerve of thigh communicated with both tibial and common peroneal nerve via communicating branches. The communicating branch with the common peroneal nerve is already mentioned above. The communicating branch with the tibial nerve arose from two rami of the tibial nerve and communicated with posterior cutaneous femoral nerve of the thigh after piercing the anomalous muscle present between these two nerves, as depicted in Figure 1B-1C.

**Figure 2:**
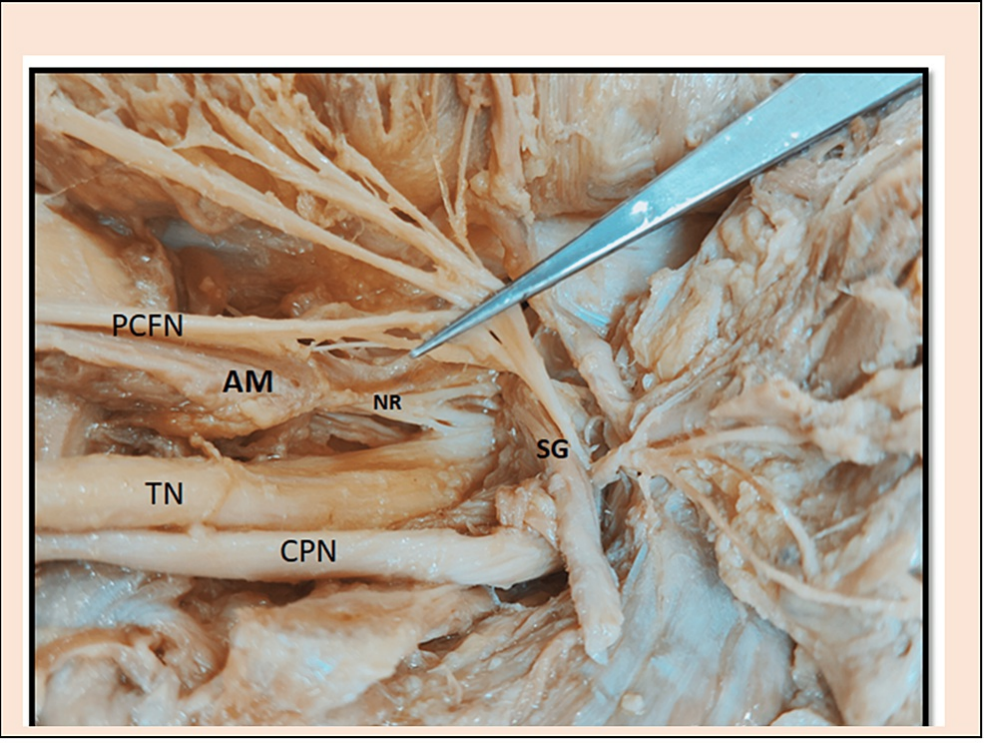
Nerve rami (NR) from the tibial nerve supplying anomalous muscle PCFN: Posterior Cutaneous Femoral Nerve; AM: Anomalous Muscle; TN: Tibial Nerve; CPN: Common Peroneal Nerve; SG: Superior Gamellus: NR: Nerve Rami

This anomalous muscle, when traced, was found to take origin from the greater sciatic notch and traveled between tibial nerve and posterior cutaneous femoral nerve of thigh, superficial to superior gemellus and seemed to insert on the ischial tuberosity in union with the origin of biceps femoris muscle. This muscle was supplied by three rami arising from the tibial nerve. The most proximal ramus took origin by two roots from the tibial nerve, traversed through anomalous muscle and communicated with PCFN. Remaining two rami arising from tibial ended by supplying the anomalous muscle, as shown in Figure 2.

## Discussion

**Figure 3:**
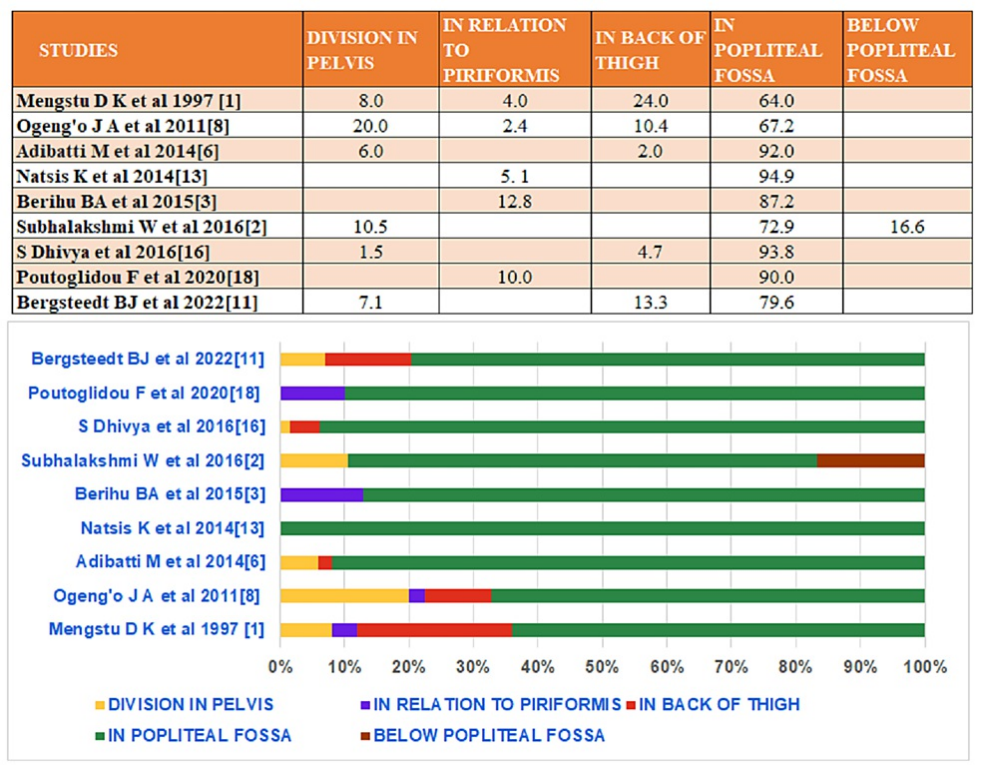
Showing frequency of division of sciatic nerve at different levels in various studies

Sciatic nerve (SN) is one of the most commonly injured nerves of the lower limb. A 0.6% stretch can also cause injury. The variations in the division of sciatic nerve are not uncommon. Division in varied regions like pelvic region, gluteal region, mid of back of the thigh, the apex of the popliteal fossa and below popliteal fossa are mentioned in the literature (Figure 3).

Bifurcation of the sciatic nerve has already been found in the pelvic region, in relation to piriformis in the gluteal region, in the posterior compartment of the thigh and below popliteal fossa. The high division of sciatic nerve in the gluteal region is more common in relation to piriformis, unlike present case where this high division was found in relation to superior gemellus. However, the present case is similar to only one case observed in 2003 by Babinski et al., where division of the sciatic nerve in relation to superior gemellus was observed wherein tibial nerve passed deep to the superior gemellus while common peroneal nerve passed superficial to the superior gemellus [9]. As depicted in Table 1, usually the sciatic nerve gets bifurcated at the superior angle of the popliteal fossa; however, very few cases mention the division of nerve below popliteal fossa (Figure 3). 

In some cases, the reunification of terminal branches of the sciatic nerve has also been found [4,8,11]. Few cases of reported trifurcation of sciatic nerve have also been observed [13-19]. Many classifications of origin and exit of sciatic nerve have been proposed in the literature. Beaton’s and Anson’s [13] original classification system is still considered the main basis for classification today. The two broad groups of variations are variants where the SN exits into the gluteal region as a common trunk or variants where the SN exits into the gluteal region pre-divided into the CPN and TN. When trying to classify, present variation does not fit into any of the categories of Beaton’s and Anson. Classifications proposed till date classify the variations of the origin of the sciatic nerve in relation to piriformis [3,13,18]. In this case, the variation was in relation to superior gemellus muscle. This encourages modifications in the present classification system.

To the best of our knowledge, the anomalous communicating branches of PCFN with tibial and common peroneal nerve and the presence of an anomalous muscle originating from the greater sciatic notch and inserting at ischial tuberosity have not been reported from reviewed literature. This anomalous muscle can be named as 'Sciaticotuberosus' after its origin and insertion.

The reported cases of a communicating branch between tibial and common peroneal nerve, presence of double piriformis and double superior gemellus and variations in the level of bifurcation of sciatic nerve related to variations in the formation of sural nerve have been observed in the literature [8,15,16].

In this case report, the bilateral higher division of sciatic nerve was found in the pelvis. Such higher division can make it more susceptible to piriformis syndrome, non-discogenic sciatica, and popliteal fossa block failure leading to local anesthesia toxicity and blood vessel traumatization. The iatrogenic injuries during hip arthroplasty and similar surgeries of the gluteal region and inadvertent damage during varicose veins stripping have also been mentioned [19,20].

Complete sciatic nerve palsy leading to loss of sensation of the posterior thigh, whole leg, and foot is uncommon, but due to anatomical reasons, common peroneal nerve injury is common manifesting as foot drop and high stepping gait [15,18]. Lower division and trifurcation of the sciatic nerve may be advantageous to surgeons doing popliteal block anesthesia but may interfere in knee surgery. Knowledge of the unusual variety of sciatic nerve enables the surgeon to find and preserve the nerve during fasciotomy, neurolysis, neuroma resection, and nerve grafting. MRI remains the gold standard imaging method for sciatic nerve variant identification to avoid intraoperative injuries.

The anomalous communicating branches of the posterior cutaneous femoral nerve (PCFN) with tibial nerve, common peroneal nerve, and its branches to superior gemellus, gluteus maximus, and the anomalous muscle make the PCFN more susceptible to nerve entrapment, PCFN neuralgia or paresthesia to one or more of its collateral branches secondary to sciatic neuropathy like piriformis syndrome. Gluteal surgeries or injections performed to alleviate sciatic neuropathy or pudendal / inferior cluneal neuralgia may risk damaging small unidentified PCFN branches due to injection site, incision placement, or accidental rupture during surgery. These anomalous branches may also play a role in idiopathic muscular atrophy [17]. The anomalous muscle is an aberrant anatomical variation that may lead to idiopathic nerve entrapment, and the possible presence of this anomalous muscle should be kept in mind by surgeons during surgeries of the gluteal region while treating buttock pain.

This variation of the sciatic nerve can co-relate with embryology. During embryological development, at the base of the limb bud, the nerves contributing to the lower limb form two plexuses (lumbar and sacral). Later, as the elements from each of these plexuses grow out into the limb, they are subdivided into dorsal and ventral components, for the dorsal and ventral musculatures. The sciatic nerve is formed when the large dorsal component of the sacral plexus (common fibular nerve) and the ventral component (tibial nerve) move downward close together during the sixth week of the embryonic stage [15,17].

The separate (autonomous) development of the sciatic nerve's tibial and common peroneal divisions could explain the source of sciatic nerve variants during embryonic development. The reason for the sciatic nerve division in the pelvis and PCFN giving anomalous branches could be certain kinds of minor mutations in the proteins involved in the molecular regulation of differentiation of dorsal (sensory) and ventral (motor) regions in developing spinal cord of the fetus. These include Transforming Growth Factor β (TGF β), Sonic Hedgehog (SHH), Bone Morphogenetic Proteins (BMP) 4 and 7, PAX3, PAX6, NKX2.2, and NKX6.1.

The patterning of muscles is determined by connective tissue derived from lateral plate mesoderm. Variations in this connective tissue development may cause the formation of anomalous muscles due to variations in muscular patterning. This could be due to some minor defects in transcription factors called Myogenic Regulatory Factors (MRFs) that activate pathways for muscle development.

## Conclusions

The current case report presents rare variation of the sciatic nerve in relation to the superior gemellus muscle. All the current classifications in literature are based on the relation of the sciatic nerve and its branches in relation to the piriformis muscle. This demands revision or modification of the current classification. A new category of division of sciatic nerve in relation to superior gemellus can be added. We also found communication of the Posterior Cutaneous Femoral Nerve (PCFN) with the tibial and common peroneal nerve. Such communication in the gluteal region is very rare and mentioned in only one case in the literature. The presence of anomalous muscle supplied by the tibial nerve rami and PCFN makes our case very unique. This anomalous muscle can be named as 'Sciaticotuberosus' after its origin and insertion. Such combinations of so many variations in one case is very rare, quite significant, and hold immense clinical significance. 
